# Social determinants of stroke prevalence in the United States adults: Analysis of 42 states using behavioral risk factor surveillance system 2022 data

**DOI:** 10.1016/j.pmedr.2025.103363

**Published:** 2025-12-26

**Authors:** Minhazul Abedin, Fazlay S. Faruque, Thomas Dobbs, Benjamin H. Walker

**Affiliations:** a Department of Population Health Science, John D. Bower School of Population Health, University of Mississippi Medical Center, 2500 N. State Street, Jackson 39216, United States; b Geospatial Health Research Lab, John D. Bower School of Population Health, University of Mississippi Medical Center, Jackson, MS 39216, United States; c Department of Preventive Medicine, John D. Bower School of Population Health, Jackson, MS 39216, United States

**Keywords:** Social determinants of health, Stroke prevalence, Comorbidity, Multimorbidity, Health disparities, Behavioral risk factor surveillance system (BRFSS)

## Abstract

**Objective:**

Social determinants of health (SDOH) significantly influence stroke outcomes and impose substantial cost burdens on the US health system. This study examined the composite effect of SDOH on prevalent stroke beyond clinical factors, highlighting comorbidity and structural inequalities using a statewide representative survey.

**Methods:**

232,155 adults across 42 U.S. states were analyzed using the 2022 Behavioral Risk Factor Surveillance System (BRFSS). A composite index of SDOH was developed based on adverse exposures. A series of logistic regression models adjusting for sociodemographic, behavioral, and health-related covariates estimated the association between the SDOH index and prevalent stroke. An additional model examined the comorbid conditions. Weighted estimates were used, with 95 % CI and a significance level of *p* < 0.05.

**Results:**

The age-adjusted prevalence ranged from 3.44 % among those with one adverse SDOH to 9.03 % among those with four or more exposures. Four or more SDOH were associated with prevalent stroke (OR: 1.44, 95 % CI: 1.23, 1.68) in the fully adjusted logistic regression model. Multimorbidity was associated with 3.52 times higher odds of prevalent stroke (95 % CI: 3.11, 3.98).

****Conclusion**:**

Adverse exposures to ≥4 SDOH significantly predicted prevalent stroke among U.S. adults. Addressing causal pathways involving multimorbidity in the SDOH-stroke relationship is critical for targeted interventions.

## Highlights

• Stroke incidents impose a costly burden on the United States health system.
• Social determinants of health play a key role in predicting stroke prevalence.
• Across 42 states, approximately 4 in 100 adults experience a stroke.
• Four or more social determinants of health significantly increase stroke risk.
• Multimorbidity and multiple comorbid conditions substantially increase stroke risk.

## 1 Introduction

Stroke is a leading cause of morbidity, mortality, and disability in the United States (U.S.), impacting approximately 795,000 individuals each year (Centers for Disease Control and Prevention, 2024). The high incidence of stroke places a significant burden on the U.S. healthcare system, with direct and indirect costs reaching up to $49.8 billion each year (Virani et al., 2020). The annual incidence of stroke mortality was estimated at 39.5 per 100,000 population in the United States (Multiple Cause of Death Data on CDC WONDER, 2025), which has recently increased after a long-term decreasing trend from 1975 to 2019 (Ananth et al., 2023). For the lower socioeconomic population, the stroke-induced higher cost of treatment and managing livelihoods is a dual challenge (Marshall et al., 2015).

The social determinants of health (SDOH) are non-medical factors influencing health outcomes, encompassing birth conditions, growth, work, life, and aging, shaped by broader economic, social, and political systems and policies (Social determinants of health, n.d). The role of social determinants of health in chronic disease is a well-established phenomenon, and they play an essential role in disease disparities, access to healthcare, and health outcomes across socioeconomic and racial or ethnic groups (Hacker et al., 2024; Kangas et al., 2025). Evidence suggests that SDOH may account for 30–50 % of an individual's health outcomes (Hood et al., 2016). While many studies have explored the impact of adverse exposure to SDOH on stroke, only a few have examined the cumulative burden of multiple adverse exposures, especially using nationally representative data in the US (Ananth et al., 2023, Reshetnyak et al., 2020, Yadav et al., 2022).

Most studies have evaluated the individual measures of social determinants of health on specific health outcomes. However, it is essential to note that these determinants are highly correlated and often occur together as a combined effect on health outcomes (Bogard et al., 2023). Few studies have examined the combined impact of social determinants of health (SDOH) on individual health outcomes, mainly using data from nationally representative surveys in the U.S. (Nelson, 2021; Zhang et al., 2024). In a large registry-based cohort study, patients from lower socioeconomic groups experienced strokes a median of 7 years earlier than their higher socioeconomic counterparts (Bray et al., 2018). A systematic review and meta-analysis of evidence suggested that low income, education, occupation, and composite measures of SDOH were associated with an increased risk of stroke mortality (Wang et al., 2020). Previous research has identified an independent association between stroke and an increased number of social determinants of health (SDOH) among individuals aged 75 and older, particularly Black women living in under-resourced neighborhoods in the Southern United States. Even after accounting for potential confounding variables, the risk of stroke was found to be 50 % higher for those experiencing three or more adverse SDOH compared to those with no SDOH (Reshetnyak et al., 2020).

The current study draws on the Behavioral Risk Factors Surveillance System (BRFSS), a statewide, nationally representative survey of adults in the U.S. The most recent survey introduced an optional module, “Social Determinants of Health and Health Equity, “ which was utilized by 42 U.S. states. Given the high interrelatedness of many social risk factors and experiences, using a composite measure of Social Determinants of Health (SDOH) proves more significant and practical than concentrating on individual social risk factors when assessing their impact on health outcomes. Recent studies have analyzed the health impact through either individual SDOH metrics (Skolarus et al., 2020, Yadav et al., 2022) or by summarizing SDOH through a cumulative count of the factors (Nelson, 2021; Zhang et al., 2024). We hypothesize that higher cumulative SDOH burden is associated with higher stroke prevalence, independent of traditional sociodemographic and clinical covariates. The study aims to estimate the stroke prevalence in 42 U.S. states as well as measure the effect of combined SDOH on prevalent stroke among adults using the 2022 BRFSS dataset. The study findings will help population scientists understand the combined effect of SDOH on stroke and design effective population-based interventions.

## 2 Materials and methods

### 2.1 Study design and population

This study utilized data from the 2022 (BRFSS), operated by the Centers for Diseases and Control (CDC) in the United States. The BRFSS is a nationally representative, statewide survey that includes non-institutionalized U.S. adults (18 years and older) using a telephone-based survey across the 50 states, the District of Columbia, and territories. The detailed methodology of the survey design and data collection can be found elsewhere ((CDC - 2022 BRFSS Survey Data and Documentation, 2025). The study population comprised all non-institutionalized U.S. adults aged 18 years and older. The overall response rate in 2022 was 45 %. The state sample selection was based on the “Social Determinants of Health and Health Equity” module, which was utilized by 42 U.S. states. The total number of respondents was 232,155 after listwise deletion of missing data. BRFSS is a publicly available dataset. Thus, no additional ethical approval was necessary for this study.

### 2.2 Measures

#### 2.2.1 Outcome

The outcome variable was the stroke status of an adult. The BRFSS asked a person, “Ever told you had a stroke?” The questionnaire was based on self-reported information from the participants, which included “No,” “Yes,” “Do not know/not sure,” and “Refused.” The analysis converted this into a binary variable consisting of the history of self-reported stroke as “Yes” or no history of stroke as “No.”

#### 2.2.2 Exposure

The BRFSS introduced Social Determinants of Health components in 2017 and developed an optional “Social Determinants of Health and Health Equity” module in 2022 containing 10 individual-level determinants based on the Center for Medicare and Medicaid Innovation Social Needs Assessment Tool, addressing employment and economic stability, housing stability and quality, food security, transportation access, utility security, loneliness, social and emotional support, life satisfaction, and mental well-being. The categories of the ten different variables under the module are listed in Table 1 in the Supplement. In 2022, 42 U.S. states utilized this module with varying questionnaire versions (Nebraska and Ohio used version 1; Oklahoma used version 2; Maryland and Michigan used split surveys with four versions combining versions 1 and 2). Following CDC guidelines and previous studies, we used summary measures of SDOH by recoding each item as binary and summing them to create an index of total SDOH impacts per participant. Participants who responded “do not know/not sure,” refused to answer, or had missing responses were classified as missing. The SDOH adverse exposure was categorized into five groups: “No SDOH,” “1 SDOH,” “2 SDOH,” “3 SDOH,” and “4 or more SDOH,”.

**Table 1:** Descriptive analysis of demographic characteristics of the adult respondents in 42 U.S states from Behavioral Risk Factor Surveillance System 2022 data (*N* = 232,155).

Variable	n (%)	Weighted % (95 % CI)
**Age group**		
18–44 years	69,422 (29.90)	44.97 (44.54, 45.40)
45–64 years	80,762 (34.79)	33.14 (32.74, 33.54)
65+ years	81,971 (35.31)	21.90 (21.59, 22.21)
**Sex**		
Female	116,924 (50.36)	48.85 (48.42, 49.28)
Male	115,231 (49.64)	51.15 (50.72, 51.58)
**Race**		
White (non-Hispanic)	180,695 (77.83)	60.43 (59.99, 60.88)
Black (non-Hispanic)	16,497 (7.11)	11.36 (11.07, 11.65)
Hispanic	17,728 (7.64)	16.30 (15.90, 16.70)
Other race	17,235 (7.42)	11.91 (11.58, 11.26)
**Marital Status**		
Never married	47,402 (20.42)	27.52 (27.12, 27.91)
Married	125,747 (54.17)	52.90 (52.47, 53.33)
Divorced/Widowed/Separated	59,006 (25.42)	19.58 (19.27, 19.90)
**Income**		
<$15,000	11,842 (5.10)	5.69 (5.48, 5.91)
$15,000 to $25,000	20,639 (8.89)	9.28 (9.02, 9.54)
$25,000 to $35,000	26,840 (11.56)	12.18 (11.90, 12.47)
$35,000 to $50,000	30,810 (13.27)	12.81 (12.52, 13.09)
Over $50,000	142,024 (61.18)	60.04 (59.62, 60.46)
**Education**		
<High school	10,397 (4.48)	9.15 (8.83, 9.48)
High School	52,650 (22.68)	25.92 (25.54, 26.30)
Some college	64,534 (27.80)	31.90 (31.49, 32.31)
College	104,574 (45.04)	33.03 (32.67–33.40)
**BMI**		
Underweight	64,702 (27.87)	28.58 (28.19, 28.98)
Normal	3263 (1.41)	1.63 (1.52, 1.75)
Overweight	83,003 (35.75)	34.66 (34.26, 35.07)
Obese	81,187 (34.97)	35.12 (34.72, 35.53)
**Health Insurance**		
No	11,283 (4.86)	7.84 (7.59, 8.11)
Yes	220,872 (95.14)	92.16 (91.89, 92.41)
**Primary care provider**		
No	28,241 (12.16)	16.35 (16.35, 17.04)
Yes	203,914 (87.84)	82.96 (82.96, 83.65)
**Smoked at least 100 cigarettes in a lifetime**		
No	203,123 (87.49)	86.28 (86.28, 86.85)
Yes	29,032 (12.51)	13.43 (13.15, 13.72)
**Exercise in the last 30 days**		
No	50,954 (21.95)	21.79 (21.44, 22.15)
Yes	181,201 (78.05)	78.21 (77.85, 78.56)
**Multimorbidity**		
No	137,254 (59.12)	64.55 (64.15, 65.94)
Yes	94,901 (40.88)	35.45 (35.06, 35.85)

#### 2.2.3 Covariates

The study included a range of covariates to capture demographic, socioeconomic, and health-related factors. Age groups were divided into “18–44 years”, “45–64 years”, and “65 years and older”. Participants were categorized by gender as “female” or “male” and by race as “White (non-Hispanic),” “Black (non-Hispanic),” “Hispanic,” and “Other race.” Marital status was classified into “Never married,” “Married,” and “Divorced/widowed/separated.” Income levels were divided into five brackets: “Less than $15,000”, “$15,000 to $25,000”, “$25,000 to $35,000”, “$35,000 to $50,000”, and “Over $50,000”. Education levels were categorized as “Less than high school,” “High school graduate,” “Some college,” and “College graduate.” Health insurance status was noted as either “Yes” or “No,” and the presence of a primary care provider was recorded as “Yes” or “No.” Smoked 100 cigarettes in a lifetime was categorized as “Yes” or “No.” In contrast, exercise in the last 30 days was noted as “Yes” and “No.” Body mass index (BMI) was categorized as “Underweight,” “Normal,” “Overweight,” and “Obese,” as per the CDC standard measure. Comorbidities include depression, chronic kidney disease, asthma, heart attack, COPD or emphysema, coronary heart disease, cancer, arthritis, diabetes, and disability (having at least one condition), which were presented as “Yes” and “No.” Lastly, multimorbidity was defined as having two or more chronic conditions (“Yes” or “No”).

#### 2.2.4 Statistical analysis

The overall and statewide weighted prevalence of stroke was estimated considering the complex survey design of BRFSS. Descriptive statistics were presented in frequency, percentage (weighted), and a Wald-type 95 % confidence interval. The estimates were disaggregated by exposure and covariates. The bivariate analysis was conducted between the outcome and predictors using the Chi-square test. In prediction, a series of Multiple Logistic Regression models was estimated, progressively adjusting for additional sets of covariates to predict the odds of each individual. Model 1 was unadjusted; Model 2 added sociodemographic variables such as age groups, marital status, education, income, health insurance, and primary care provider; Model 3 further added history of smoking 100 cigarettes in a lifetime, exercise in last 30 days, and Model 4 included the indicator of multimorbidity. Next, an additional model was estimated using each morbidity as a predictor in the model rather than the multimorbidity indicator. The multicollinearity assumption was verified using Variance Inflation Factors (VIF). All data were analyzed using STATA 19.0 between 2024 and 2025.

## 3 Results

Table 1 represents the demographic and socioeconomic characteristics of the respondents in our analysis. The total number of eligible respondents for this study was 232,155. Of these respondents, 50.4 % (*n* = 116,924) were female. Around 35 % (*n* = 81,971) of the respondents were 65 years and older. About 79 % (*n* = 180,695) were non-Hispanic white. Only 7 % (*n* = 16,497) were black African American, and around 8 % (*n* = 17,728) were Hispanic. The weighted prevalence of overweight and obesity was 34.7 % (95 % CI: 34.26, 35.07) and 35.1 % (95 % CI: 34.72, 35.53), respectively. Estimated weighted multimorbidity was 34.72 % (95 % CI: 34.34, 35.10). A separate descriptive table for each comorbidity has been provided in Table 3 in the Supplement.

The overall weighted prevalence of stroke was 3.5 % (95 % CI: 3.31 %, 3.61 %) for the selected 42 U.S. states, presented in Table 2. The age-adjusted prevalence was calculated for groups based on the cumulative number of SDOH experienced by each adult individual. The estimates ranged from 2.56 % for those without adverse exposure to SDOH to 9.0 % for those with four or more adverse SDOH exposures.

**Table 2:** Prevalence estimates of stroke by social determinants of health among adults in 42 U.S states from Behavioral Risk Factor Surveillance System 2022 data (N = 348,778).

	Unweighted N	Weighted N	Weighted Prevalence (95 % CI)	Age-Adjusted Prevalence
Overall Sample	9682	4,618,404	3.45 (3.31, 3.61)	–
No SDOH	4574	2,089,471	2.79 (2.63, 2.97)	2.62 (2.45, 2.79)
1 SDOH	1881	873,801	3.56 (3.16, 4.03)	3.44 (3.02, 3.85)
2 SDOH	1139	511,274	3.76 (3.37, 4.20)	4.36 (3.89, 4.83)
3 SDOH	696	323,298	4.16 (3.57, 4.86)	5.32 (4.53, 6.12)
4 or more SDOH	1392	820,560	6.27 (5.65, 6.95)	9.03 (8.14, 9.92)

Table 3 presents the results of multiple logistic regression models examining the impact of SDOH on stroke outcomes in adults. Model 1 was a single-variable logistic model, where any exposure to SDOH was highly significant in predicting stroke risk, with a greater association observed in those with four or more SDOH. Model 2 was expanded to include age, race, marital status, education, income, health insurance, BMI, and primary care provider as additional covariates. This model showed only minor attenuation in the association between SDOH and stroke. In model 3, we included smoking status (smoked at least 100 cigarettes in a lifetime) and exercise in the last 30 days. This model showed that exposure to at least one or more SDOH was significantly associated with prevalent stroke. As shown in Model 4, after adjusting for multimorbidity status, only exposure to 4 or more SDOH was significantly associated with the prevalent stroke (OR: 1.4, 95 % CI: 1.23, 1.68). Respondents with more than one comorbid condition had 3.6 times greater odds of prevalent stroke than those with no or one comorbid condition (OR: 3.5, 95 % CI: 3.11, 3.98).

**Table 3:** Multiple logistic regression models predicting the stroke risk among adults in 42 U.S states using Behavioral Risk Factor Surveillance System 2022 data (N = 348,778).

**Variable**	Model 1 (OR)	Model 2 (OR)	Model 3 (OR)	Model 4 (OR)
**Summary measure of SDOH**				
No SDOH	**Reference**			
1 SDOH	1.29*** (1.12, 1.48)	1.19* (1.03, 1.38)	1.16* (1.00, 1.34)	1.10 (0.95, 1.28)
2 SDOH	1.36*** (1.19, 1.55)	1.31*** (1.14, 1.51)	1.25** (1.09, 1.44)	1.09 (0.95, 1.25)
3 SDOH	1.51*** (1.27, 1.80)	1.42*** (1.19, 1.70)	1.32** (1.10, 1.58)	1.08 (0.90, 1.30)
4 or more SDOH	2.33*** (2.05, 2.64)	2.15*** (1.84, 2.52)	1.95*** (1.66, 2.29)	1.44*** (1.23, 1.68)
**Age groups**				
18–44 years	**Reference**			
45–64 years		3.59*** (2.94, 4.38)	3.39*** (2.78, 4.13)	2.86*** (2.34, 3.51)
65 years or older		6.35*** (5.18, 7.79)	6.12*** (5.01, 7.49)	4.21*** (3.40, 5.22)
**Sex**				
Female	**Reference**			
Male		1.24* (1.12, 1.37)	1.25** (1.05, 1.38)	1.30*** (1.15, 1.52)
**Race**				
White (non-Hispanic)	**Reference**			
Black (non-Hispanic)		1.19* (1.03, 1.36)	1.21** (1.05, 1.38)	1.32** (1.15, 1.52)
Hispanic		0.70** (0.56, 0.89)	0.74** (0.58, 0.93)	0.85 (0.67, 1.07)
Other race		0.98 (0.83, 1.17)	1.00 (0.84, 1.19)	1.04 (0.87, 1.24)
**Marital Status**				
Never married	**Reference**			
Married		1.34*** (1.14, 1.58)	1.35*** (1.15, 1.58)	1.33*** (1.13, 1.56)
Divorced/Widowed/Separated		1.56*** (1.32, 1.84)	1.52*** (1.29, 1.80)	1.48*** (1.16, 1.75)
**Education**				
<High school		1.71*** (1.39, 2.11)	1.50*** (1.22, 1.86)	1.37*** (1.11, 1.70)
High School		1.29*** (1.13, 1.47)	1.23* (1.03, 1.35)	1.16 (0.99, 1.30)
Some college		1.30*** (1.13, 1.46)	1.23*** (1.09, 1.38)	1.16* (1.03, 1.30)
College	**Reference**			
**Income**				
<$15,000		2.72*** (2.24, 3.24)	2.49*** (2.05, 3.03)	2.09*** (1.72, 2.53)
$15,000 to $25,000		2.31*** (1.96, 2.73)	2.17*** (1.84, 2.57)	1.85*** (1.57, 2.18)
$25,000 to $35,000		2.04*** (1.74, 2.38)	1.93*** (1.65, 2.26)	1.70*** (1.45, 1.99)
$35,000 to $50,000		1.55*** (1.32, 1.81)	1.50*** (1.28, 1.75)	1.39*** (1.19, 1.62)
Over $50,000	**Reference**			
**Health insurance**				
Yes	**Reference**			
No		0.89 (0.71, 1.13)	0.89 (0.70, 1.13)	0.98 (0.77, 1.24)
**Primary care provider**				
Yes	**Reference**			
No		0.51*** (0.42, 0.61)	0.50*** (0.41, 0.59)	0.59*** (0.49, 0.70)
**BMI**				
Underweight		1.12(0.71, 1.76)	1.04 (0.67, 1.61)	1.00 (0.64, 1.56)
Normal	**Reference**			
Overweight		0.93 (0.82, 1.06)	0.93 (0.82, 1.06)	0.88 (0.78, 1.00)
Obese		1.04 (0.92, 1.07)	1.01 (0.90, 1.15)	0.86* (0.76, 0.98)
**Smoked at least 100 cigarettes in a lifetime**				
No	**Reference**			
Yes			1.35*** (1.20, 1.51)	1.24*** (1.10, 1.39)
**Exercise in the last 30 days**				
Yes	**Reference**			
No			1.55*** (1.40, 1.70)	1.39*** (1.26, 1.53)
**Presence of multimorbidity**				
No	**Reference**			
Yes				3.52*** (3.11, 3.98)

* p < 0.05; ** *p* < 0.01; *** *p* < 0.001.

**Fig. 1:**
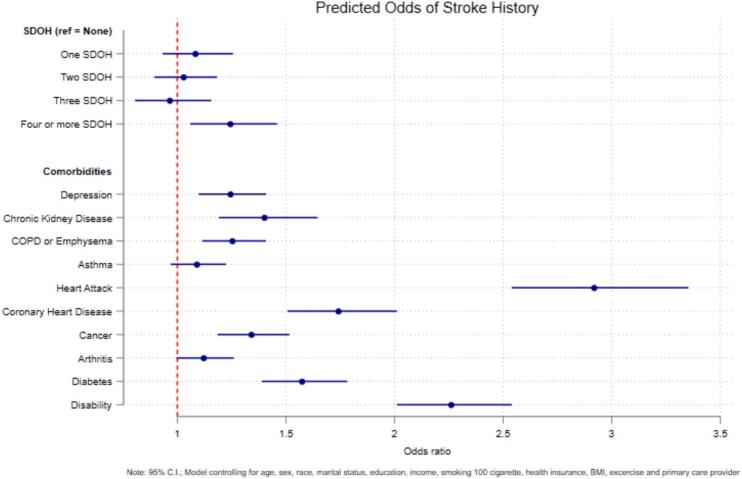
Predicted odds of stroke history by social determinants of health and comorbidities among adults in 42 U.S states using Behavioral Risk Factor Surveillance System 2022 data.

An additional set of models was estimated, including each comorbid condition in the analysis, with the same progressive adjustment strategy and the same covariates as those included in Table 3. Before model estimation, multicollinearity was checked among the comorbidities, and the mean Variance Inflation Factor (VIF) was 1.31, indicating that the model was not significantly affected by multicollinearity. The results of the fully adjusted model are shown in Fig. 1. These results suggest that an adult with exposure to 4 or more SDOH had 1.2 times the odds of sustaining a stroke compared to those without exposure to any SDOH (95 % CI: 1.06, 1.46). All the comorbid conditions except for arthritis and asthma significantly predicted the stroke risk among U.S. adults. The comorbid conditions with the greatest association included heart attack (OR: 2.9, 95 % CI: 2.54, 3.35), disability (OR: 2.3, 95 % CI: 2.01, 2.54), and coronary heart disease (OR: 1.7, 95 % CI: 1.51, 2.01).

## 4 Discussion

The study investigated the relationship between the combined measure of SDOH and stroke prevalence in a large sample of U.S. adults in 2022, and highlighted the significant role that SDOH and multimorbidity play in shaping stroke outcomes.

Our findings suggest that the increasing number, particularly four or more SDOH, significantly predicts a higher odds of stroke. Similar findings have been reported in previous research on stroke and multiple SDOH in the adult U.S. population (Faigle and Towfighi, 2024, Yadav et al., 2022, Yang et al., 2025). This association remained significant even after adjusting for key demographic and socioeconomic covariates, including age, race, income, education, BMI, and access to healthcare. Although increasing age was a significant predictor of higher stroke risk for adults aged 45–64 years and those over 65 compared to the 18–44 years adults, strokes among young adults are currently increasing, and non-traditional risk factors are appearing significantly, besides the traditional factors (Leppert et al., 2024).

Consistent with previous research, multimorbidity significantly heightened the odds of stroke, with individuals having two or more chronic conditions facing more than three times the odds of stroke compared to those without or at least one comorbidity. While mediation was not directly tested, the results suggest that the SDOH-stroke relationship may be partially mediated through comorbidities. These results further solidify the well-documented connection between chronic disease and stroke risk, multimorbidity as an independent risk factor for stroke, which often leads to long-term disability and mortality (Downer et al., 2024; Gallacher et al., 2019; Kelly and Rothwell, 2021).

Individuals exposed to adverse social conditions are more likely to develop multimorbidity, increasing their stroke risk (Álvarez-Gálvez et al., 2023; Mossadeghi et al., 2023). The higher odds of multimorbidity in the fully adjusted model underscore the importance of addressing social and health-related factors in population health strategies to control multimorbidity and reduce stroke prevalence. Interventions aimed at mitigating the adverse impacts of SDOH, such as improving access to healthcare, reducing income inequality, and enhancing education opportunities, may also indirectly reduce multimorbidity and, by extension, stroke risk (Sur et al., 2024). Our findings show that certain conditions, including heart attack, disability, and coronary heart disease, were significantly associated with increased stroke risk, while others, like arthritis and asthma, were not (Aparicio, 2019; Sadeq et al., 2022). Previous research suggests that black U.S. adults are at a higher risk of stroke compared to their white counterparts. The risk is significantly higher in young and middle-aged adults (Madsen et al., 2024). A trend analysis from 1999 to 2019 showed that non-Hispanic black African Americans experienced a higher risk of mortality from stroke compared to non-Hispanic white adults (Yang et al., 2023). This shows a long-term disparity in racial health outcomes, not only for stroke morbidity and mortality but also for other chronic conditions in U.S. adults. Our research found a 20 % decreased risk of stroke among Hispanic adults. A possible reason for their decreased risk could be the “Hispanic Paradox” or the unexplained mediating factors in the model (Hernandez et al., 2022).

The strong association between SDOH and stroke points to the need for integrated approaches to stroke prevention. Policies that address social determinants, such as improving access to healthcare, reducing poverty, and fostering educational attainment, are essential to reducing stroke risk, particularly among socially disadvantaged populations. The persistence of SDOH effects, even after adjusting for individual-level health behaviors like smoking and exercise, suggests that addressing these behavioral risk factors alone will not be sufficient to mitigate stroke disparities at the optimum level. Public health efforts must focus on broader structural determinants to improve social conditions and reduce health inequities.

The study's strength lies in its large and diverse sample, which allowed robust analysis of SDOH, multimorbidity, and stroke across various demographic groups. However, limitations include the cross-sectional design limiting to infer causal inference; potential unmeasured confounders (Ananth and Schisterman, 2018); lack of individual SDOH type reporting, as the focus was on the cumulative measures, and reliance on self-reported responses for stroke status through a yes/no question, which may introduce reporting bias despite reasonable validity shown in previous epidemiological studies (Miilunpalo et al., 1997, Pierannunzi et al., 2013). An important limitation of our study is the use of a cumulative SDOH index that counts adverse exposures without distinguishing between different types or combinations of social determinants. The index captures the overall burden of social disadvantage but not domain-specific pathways or configurations of stroke prevalence. This approach assumes equal weighting across SDOH domains and cannot identify which specific combinations of SDOH domains most strongly drive stroke risk. Different SDOH likely influence stroke through distinct causal pathways. By aggregating these heterogeneous factors into a single count, we may obscure meaningful variation in how different SDOH patterns influence stroke prevalence.

Future research should consider the longitudinal study design to investigate the causal pathways between the SDOH and stroke in U.S. adults, particularly among the vulnerable population in the stroke belt. Given multimorbidity's substantial influence, decomposition analysis and latent class analysis should be used to estimate individual comorbidity contributions and identify distinct SDOH patterns.

## 5 Conclusion

There exist significant disparities in stroke morbidity among U.S. adults. Multiple social determinants of health, specifically ≥4 SDOH, strongly predict stroke prevalence. Individual comorbidities and multimorbidity appear to strongly influence the relationship between SDOH and stroke. These findings underscore the need for longitudinal studies to elucidate causal mechanisms and inform targeted population health strategies that address both social factors and their downstream health consequences.

## CRediT authorship contribution statement

**Minhazul Abedin MPH:** Writing – original draft, Methodology, Formal analysis, Data curation, Conceptualization, Writing – review & editing. **Fazlay S. Faruque:** Writing – review & editing, Validation, Supervision, Methodology, Conceptualization. **Thomas Dobbs:** Writing – review & editing, Validation, Supervision, Conceptualization, Methodology. **Benjamin H. Walker:** Writing – review & editing, Validation, Supervision, Methodology, Formal analysis, Data curation, Conceptualization.

## Declaration of competing interest

The authors declare that they have no known competing financial interests or personal relationships that could have appeared to influence the work reported in this paper.

## Data Availability

The data source used for this research is public

## Appendix A Supplementary data

Supplementary materials Supplementary materials include a sample selection flowchart, detailed variable definitions for the 2022 BRFSS social determinants of health and health equity module, state-level characteristics (N = 42), and comorbidity prevalence estimates (N = 232,155). Image 1
