# Supplementary material for: Social determinants of stroke prevalence in the United States adults: Analysis of 42 states using behavioral risk factor surveillance system 2022 data
Source: Prev Med Rep. 2025 Dec 26;61:103363. doi: 10.1016/j.pmedr.2025.103363 (PMC12808595; doi:10.1016/j.pmedr.2025.103363)
Supplement: Supplementary materials — include a sample selection flowchart, detailed variable definitions for the 2022 BRFSS social determinants of health and health equity module, state-level characteristics (N = 42), and comorbidity prevalence estimates (N = 232,155). [file mmc1.docx]

**Supplement**

Figure 1 (Supplement): Flowchart of the sample selection used for the analysis of Behavioral Risk Factor Surveillance System 2022 data based on social determinants of health and health equity module


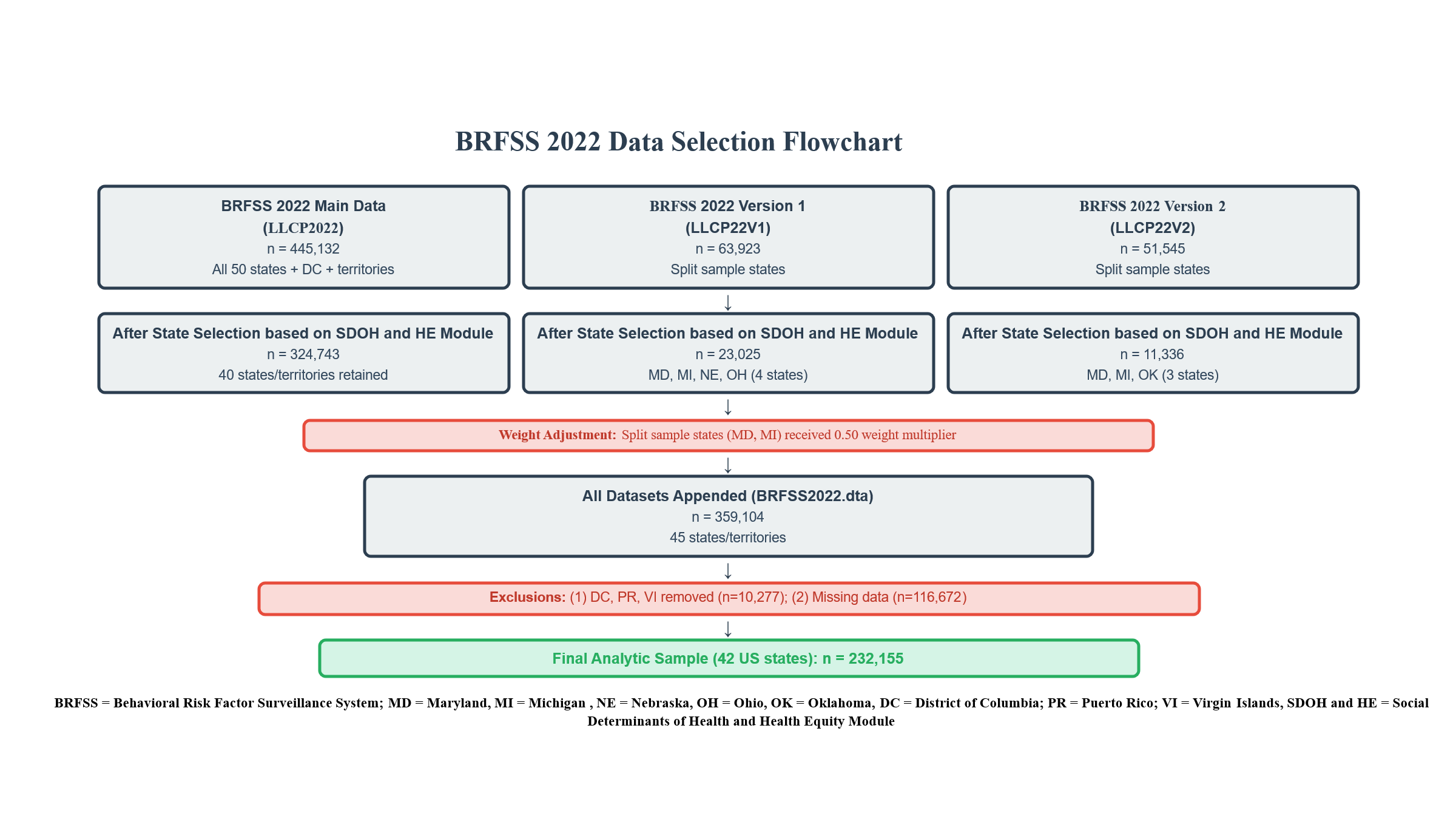


**Social determinants of health and health equity module**

The Module consisted of 10 different measures of SDOH and related well-being indicators (Centers for Disease Control, 2022). These aim to assess the specific psychosocial, economic, and health-related domains. Each indicator has the following categories:

Table 1 (Supplement): Social determinants of health and health equity module variable, categories, and objectives used for the analysis of Behavioral Risk Factor Surveillance System 2022 data

| **Indicator of SDOH** | **Categories** | **Objective** |
| --- | --- | --- |
| **Life Satisfaction** | 1. Very satisfied 2. Satisfied 3. Dissatisfied 4. Very dissatisfied 5. Don’t know/not sure 6. Refused | Assessed overall life satisfaction with response options on a four-point Likert scale |
| **Social and Emotional Support** | 1. Always 2. Usually 3. Sometimes 4. Rarely 5. Never 6. Don’t know/not sure 7. Refused | Measured the frequency of receiving needed social and emotional support. |
| **Social Isolation** | 1. Always 2. Usually 3. Sometimes 4. Rarely 5. Never 6. Don’t know/not sure 7. Refused | Evaluated how often respondents felt socially isolated. |
| **Employment Stability** | 1. Yes 2. No 3. Don’t know/not sure 4. Refused | Asked if respondents had experienced job loss or reduced hours in the past 12 months |
| **Food Security (SNAP Participation)** | 1. Yes 2. No 3. Don’t know/not sure 4. Refused | Assessed SNAP participation (use of Supplemental Nutrition Assistance Program benefits) in the last 12 months. |
| **Food Security (Inadequate Food Supply)** | 1. Always 2. Usually 3. Sometimes 4. Rarely 5. Never 6. Don’t know/not sure 7. Refused | Asked about the frequency of food shortages due to financial limitations over the past year |
| **Housing Security** | 1. Yes 2. No 3. Don’t know/not sure 4. Refused | Assessed the inability to pay for mortgage, rent, or utility bills within the last 12 months, with responses. |
| **Utility Security** | 1. Yes 2. No 3. Don’t know/not sure 4. Refused | Addressed threats of utility shutoff (electric, gas, oil, or water services) within the past year. |
| **Transportation Access** | 1. Yes 2. No 3. Don’t know/not sure 4. Refused | Assessed limitations in reliable transportation that may have prevented access to essential activities (e.g., medical appointments, work, daily necessities) over the past year. |
| **Mental Well-being** | 1. Always 2. Usually 3. Sometimes 4. Rarely 5. Never 6. Don’t know/not sure 7. Refused | Evaluated stress experiences in the past 30 days, defined as the feelings of tension, restlessness, nervousness, or anxiety affecting sleep. |

Table 2 (Supplement): State information (N=42) used for the analysis of Behavioral Risk Factor Surveillance System 2022 data

| State FIPS Code | Name of the State | N (%) | Weighted % |
| --- | --- | --- | --- |
| 1 | Alabama | 3,119 (1.34) | 4.91 (3.83, 6.26) |
| 2 | Alaska | 3,979 (1.71) | 2.99 (2.42, 3.69) |
| 4 | Arizona | 6,789 (2.92) | 3.85 (3.20, 4.62) |
| 6 | California | 6,872 (2.96) | 2.45 (2.03, 2.95) |
| 9 | Connecticut | 6,025 (2.60) | 2.65 (2.09, 3.35) |
| 10 | Delaware | 2,643 (1.14) | 3.70 (2.98, 4.58) |
| 12 | Florida | 8,469 (3.65) | 4.04 (3.34, 4.89) |
| 13 | Georgia | 5,801 (2.50) | 3.61 (2.97, 4.37) |
| 16 | Idaho | 4,364 (1.88) | 3.15 (2.56, 3.87) |
| 18 | Indiana | 6,467 (2.79) | 4.16 (3.65, 4.73) |
| 19 | Iowa | 6,427 (2.77) | 3.07 (2.59, 3.64) |
| 20 | Kansas | 7,898 (3.40) | 2.77 (2.34, 3.27) |
| 21 | Kentucky | 2,643 (1.14) | 4.02 (3.22, 5.01) |
| 23 | Maine | 7,265 (3.13) | 3.47 (2.95, 4.08) |
| 24 | Maryland | 6,926 (2.98) | 3.28 (2.77, 3.89) |
| 25 | Massachusetts | 6,933 (2.99) | 2.70 (2.23, 3.26) |
| 26 | Michigan | 4,178 (1.80) | 2.93 (2.42, 3.55) |
| 27 | Minnesota | 11,704 (5.04) | 3.05 (2.63, 3.53) |
| 28 | Mississippi | 2,957 (1.27) | 4.31 (3.46, 5.35) |
| 29 | Missouri | 5,141 (2.21) | 3.68 (3.08, 4.39) |
| 30 | Montana | 5,179 (2.23) | 3.26 (2.71, 3.92) |
| 31 | Nebraska | 2,810 (1.21) | 2.45 (1.91, 3.13) |
| 32 | Nevada | 2,186 (0.94) | 3.25 (2.40, 4.39) |
| 33 | New Hampshire | 4,319 (1.86) | 2.71 (2.24, 3.28) |
| 34 | New Jersey | 4,837 (2.08) | 2.95 (2.17, 3.99) |
| 35 | New Mexico | 3,566 (1.54) | 2.94 (2.34, .69) |
| 37 | North Carolina | 3,015 (1.30) | 4.46 (3.38, 5.86) |
| 38 | North Dakota | 2,977 (1.28) | 2.70 (2.13, 3.41) |
| 39 | Ohio | 7,173 (3.09) | 4.10 (3.50, 4.80) |
| 40 | Oklahoma | 1,947 (0.84) | 3.99 (3.18, 4.99) |
| 41 | Oregon | 3,916 (1.69) | 3.97 (3.30, 4.78) |
| 44 | Rhode Island | 3,788 (1.63) | 2.81 (2.18, 3.60) |
| 45 | South Carolina | 6,316 (2.72) | 3.65 (3.13, 4.25) |
| 47 | Tennessee | 3,539 (1.52) | 5.11 (4.28, 6.10) |
| 48 | Texas | 8,919 (3.84) | 3.67 (3.00, 4.47) |
| 49 | Utah | 6,575 (2.83) | 2.13 (1.73, 2.61) |
| 50 | Vermont | 5,957 (2.57) | 3.20 (2.52, 4.05) |
| 51 | Virginia | 6,869 (2.96) | 3.40 (2.91, 3.97) |
| 53 | Washington | 17,552 (7.56) | 2.54 (2.28, 2.82) |
| 54 | West Virginia | 3,649 (1.57) | 5.67 (4.91, 6.54) |
| 55 | Wisconsin | 7,589 (3.27) | 3.12 (2.68, 3.63) |
| 56 | Wyoming | 2,877 (1.24) | 3.14 (2.52-3.92) |
| **Total** | | **232,155 (100)** | **3.45 (3.31, 3.61)** |

Table 3 (Supplement): Comorbidity among adults in 42 U.S states using Behavioral Risk Factor Surveillance System 2022 data (N=232,155)

| **Comorbidity** | **N (%) [Yes]** | **Weighted %** |
| --- | --- | --- |
| Ever told you have a depressive disorder? | 50,908 (21.93) | 21.80 (21.47, 22.14) |
| Ever told you have a kidney disease? | 10,541 (4.54) | 3.69 (3.54, 3.85) |
| Ever told you had COPD or emphysema? | 18,533 (7.98) | 6.78 (6.58, 6.97) |
| Ever told had asthma | 35,240 (15.18) | 15.29 (14.99, 15.59) |
| Ever diagnosed with heart attack? | 12,811 (5.52) | 4.24 (4.09, 4.40) |
| Ever diagnosed with angina or coronary heart disease? | 14,209 (6.12) | 4.52 (4.37, 4.67) |
| (Ever told you had melanoma or any cancer? | 27,267 (11.75) | 8.38 (8.17, 8.59) |
| Ever told you have arthritis? | 80,512 (34.68)68 | 27.21 (26.85, 27.57) |
| Ever told you had diabetes (not gestational)? | 31,471 (13.56)56 | 12.01 (11.75, 12.29) |
| Do you have any disability? | 68,916 (29.69) | 27.99 (27.61, 28.37) |
